# Gene Polymorphism in Five Target Genes of Immunosuppressive Therapy and Risk of Development of Preeclampsia †

**DOI:** 10.3390/healthcare9070821

**Published:** 2021-06-28

**Authors:** Francesca Previtera, Stefano Restaino, Giulio Romano, Giuseppe Vizzielli, Andrea Neri, Elisa Scalzotto, Luigi Vetrugno, Beatrice Montessoro, Roberto Mioni, Lorenza Driul

**Affiliations:** 1 Obstetrics and Gynecology Unit, Department of Obstetrics Gynecology and Pediatrics, Udine University Hospital, DAME, Piazzale Santa Maria della Misericordia 15, 33100 Udine, Italy; francesca.previtera90@gmail.com (F.P.); giuseppevizzielli@yahoo.it (G.V.); dott.montessoro@gmail.com (B.M.); lorenza.driul@uniud.it (L.D.); 2 Dialysis and Transplantation Unit, Department of Nephrology, Udine University Hospital, DAME, Piazzale Santa Maria della Misericordia 15, 33100 Udine, Italy; giulio.romano@uniud.it (G.R.); roberto.mioni@asufc.sanita.fvg.it (R.M.); 3 Dialysis and Transplantation Unit, Department of Nephrology, International Renal Research Institute of Vicenza, San Bortolo Hospital, Viale Ferdinando Rodolfi 37, 36100 Vicenza, Italy; andreaneri194@gmail.com (A.N.); elisascalzotto85@gmail.com (E.S.); 4 Department of Anesthesia and Intensive Care, Udine University Hospital, Piazzale Santa Maria della Misericordia 15, 33100 Udine, Italy; luigi.vetrugno@uniud.it

**Keywords:** preeclampsia, gene polymorphism, immunosuppressive therapy, ABCB1/MDR1 gene, renal transplant

## Abstract

Pregnancy can be considered as an allogeneic transplant and preeclampsia can be seen as a failure of the acceptance mechanisms of this transplant as occurs in acute organ transplant rejection. Some genetic polymorphisms may be involved in its pathogenesis. Since the kidney is one of the organs mainly involved in preeclampsia, our study attempted to determine the frequencies of single nucleotide polymorphisms of DNA (SNP) in 3 genes (adenosine triphosphate-binding cassette sub-family B member 1 (ABCB1)/multi drug reactivity 1 (MDR1) gene, interleukin 10 gene and tumor necrosis factor α gene) which are targets of immunosuppressive therapies and related to acute renal rejection. The study was an observational, monocentric, case-control study. We enrolled 20 women with severe preeclampsia and 10 women age-matched with regular pregnancy. Continuous variables were compared by the Student’s *t*-test for independent variables or using the Mann-Whitney test depending on their distribution. We used Fisher test to compare categorical variables between cases and controls, while we used logistic regression model to evaluate which risk factor was associated with preeclampsia. Although there was no statistically significant difference between the two groups, we found different percentages of two of the polymorphisms considered (rs1045642 and rs2032582 in the gene ABCB1). Despite these results, our work may be helpful for future research to better understand the pathogenesis of preeclampsia.

## 1. Introduction

Preeclampsia is a major cause of maternal, fetal and neonatal mortality as well as maternal and neonatal morbidity. It is characterized by new-onset hypertension accompanied by new-onset proteinuria. The diagnostic criteria of preeclampsia are listed in Table 1 [1]. The incidence of preeclampsia is 3–5% of all pregnancies around the world [2] and an early detection of this disorder is critical because it may progress to eclampsia (convulsive form) and impair fetal growth.

Furthermore, the onset of this pathology can affect several organs, first of all the kidney, and some of these women can worsen up to kidney failure.

Pregnancy can be considered as an immuno-accepted allogeneic transplant. For a successful pregnancy the functional balance between T helper phenotype 1 (TH1) and T helper phenotype 2 (TH2) is crucial. Current evidence suggests that the success of fetal tolerance is based on an immunological deviation to a TH2-induced antibody response instead of predominantly TH1-induced response [3]. The tolerance of the fetus is therefore allowed by various factors: on one hand purely immunological factors and on the other immunoregulatory molecules produced by the fetusplacental unit which together contribute to the maintenance and viability of the fetal allograft [4].

In this context preeclampsia can be considered as the failure of this system and pathogenesis is related to a still unclear mechanism involving the immune system and kidney.

Until now there are no predictive tests to identify which patients will present an increased risk of developing severe preeclampsia at an early stage. Therefore, the identification of genetic profiles with greater or lesser risk of developing this syndrome may be a useful contribution.

The aim of our study was to determine the allelic and genotypic frequencies of some single nucleotide polymorphisms (SNP) of DNA in 5 genes (adenosine triphosphate-binding cassette sub-family B member 1 (ABCB1)/multi drug reactivity 1 (MDR1) gene, genes coding for interleukin 10 (IL-10) and for tumor necrosis factor α (TNF-α)), which are targets of immunosuppressive therapies used in acute rejection in kidney transplantation and could therefore be involved in the pathogenesis of preeclampsia.

## 2. Materials and Methods

### 2.1. Enrollment

This is an observational, monocentric, case-control study. The study protocol was approved by the Ethics Committee of the Udine University Hospital on July the 13th, 2016 with approval number 44/16.

We enrolled thirty patients who went to Gynecology and Obstetrics Unit of the Udine University Hospital between September 2016 and June 2017. We divided these patients into two groups: the first group (case group) was composed by twenty pregnant women who were enrolled during hospitalization for severe preeclampsia (according to the ACOG 2019 criteria listed in Table 2) [1].

The other group (control group) was composed by ten pregnant women matched for age with the case group, who had a regular pregnancy defined as pregnancy without any fetal or maternal problems that could complicate its regular course (diabetes, hypertensive disorders, cholestasis, fetal growth restriction etcetera).

All women included in this study had signed the informed consent of participation.

All data was collected in an anonymous database.

### 2.2. Genetic Analysis

For all women, we collected two peripheral blood samples (12 mL divided into two test tubes each one containing 6 mL of blood in EDTA). All blood samples were processed in the International Renal Research Institute Vicenza (IRRIV) Laboratories of the Department of Nephrology, Dialysis and Transplantation, San Bortolo Hospital Vicenza.

On the base of published literature, we analyzed 5 SNPs located in three different genes: rs1800872 in the IL-10 gene; rs1800629 in the TNF gene; rs1045642, rs1128503 and rs2032582 in the ABCB1 gene.

The genetic analysis was achieved by DNA extraction and purification, DNA qualitative and quantitative evaluation, polymerase chain reaction (PCR), agarose gel electrophoresis, fragment purification, Sanger sequencing reaction, purification of the sequences and capillary electrophoresis. DNA was extracted from each frozen blood sample using the NorDiag Arrow instrument with the Blood DNA 200 Extraction Kit disposable cartridge (DiaSorin Ireland Ltd., Dublin, Ireland). The instrument uses a magnetic bead-based extraction method. The NanoPhotometer N50 Touch (Implen, Schatzbogen, Germany) instrument, based on a spectrophotometric technology, was used for DNA quantitative and qualitative evaluation. The necessary parameters were adjusted before preparing the reaction mix for the PCR: primer design and annealing temperature (Ta). The primers were designed using the Primer3 program (Whitehead Institute, Cambridge, MA, USA), as shown in Table 3.

PCR was performed using the standardized reaction mix AmpliTaq Gold 360 MasterMix (Applied Biosystems, Foster City, CA, USA), which contains a specific Hot Start Taq polymerase enzyme. The protocol (thermal cycling) was as follows: initial denaturation: 10 min at 95 °C; 35 amplification cycles of denaturation: 30 s at 95 °C, annealing: according to the SNPs, extension: 40 s at 72 °C; final extension cycle: 10 min at 72 °C. The PCR products were verified by electrophoresis in 2% agarose gel (Starpure Agarose melted in Tris Borate EDTA buffer) stained with Midori Green Direct (Nippon Genetics, Dueren, Germany). We used Illustra Exo-ProStar 1-Step (GE Healthcare Bio-Sciences, Pittsburgh, MA, USA) for purification of the fragments, according to the instructions of the manufacturer. The Sanger sequencing reaction was performed using the Big Dye Terminator v3.1 Cycle Sequencing Kit (Applied Biosystems) according to the instructions of the manufacturer. The products of the reaction were purified using Centri-Sep Columns (Princeton Separations, Freehold, NJ, USA), according to the instructions of the manufacturer. The purified sequences were denatured using HiDi deionized formamide (Applied Biosystems): the denaturation protocol consisted of 5 min at 95 °C. At the end we analyzed the sequences using capillary electrophoresis with the AB 3500 Genetic Analyzer (Applied Biosystems). The obtained sequences were evaluated using the Variant Reporter Software v1.1 (Applied Biosystems).

### 2.3. Statistical Analysis

Continuous variables were expressed as median and Interquartile Range (IQR) or as mean ± standard deviation (SD), depending on their distribution, while categorical variables were described as frequencies. Continuous variables were compared by the Student’s *t*-test for independent variables or using the Mann-Whitney test depending on their distribution. We used Fisher test to compare categorical variables between cases and controls, while we used logistic regression model to evaluate which risk factor was associated with pre-eclampsia. For each SNP the heterozygosity (HET) was determined and the test for Hardy-Weinberg equilibrium (HWE) was performed in the control groups and in the cases by the Chi-Square Goodness-of-Fit Test. The measure of heterozygosity (HET) indicates how many individuals are heterozygous for a given locus and represents an index of genetic variability. Allelic frequencies and genotype frequencies were determined for each SNP. Genetic associations were explored using ‘per-genotype’ analysis, ‘per-allele’ analysis and ‘linear trend’ analysis using the SAS version 9.1.4 program (SAS Institute Inc., Cary, NC, USA). Statistical significance was evaluated by the two-tailed *t*-test (*p* < 0.05).

## 3. Results

Analyzing the demographic characteristics of our population, no statistically significant differences emerged between the two groups. As shown in Table 4, mean age and mean Body Mass Index (BMI) were similar.

Regarding parity in the case group the majority of women were nulliparous (12/20), while all women in the control group were not at their first pregnancy. Considering risk factors, one woman in the case group was affected by diabetes mellitus and another one in the same group by gestational diabetes. None reported familiarity with preeclampsia. In the case group only 3 patients were affected by hypertension before pregnancy. In the same group there were 3 twin pregnancies and 5 women got pregnant with assisted reproductive technologies (egg donation, Intracytoplasmic Sperm Injection or In vitro fertilization and embryo transfer).

Caesarean section was performed in 18/20 women in case group while 9/10 women in the control group had a vaginal delivery. Regarding prematurity, in the control group none delivered before 37 weeks while in the case group 16/20 women had a preterm delivery. 

Genetic analysis showed heterogeneous results as shown in Table 4. We analyzed three polymorphisms of the *ABCB1* gene: rs1045642, rs1128503 and rs2032582. We found rs2032582 mutation in 15/20 of women of the case group and 5/10 of control group; the polymorphism rs1128503 was present in 11 cases and 6 controls and rs1045642 was found in 7 cases and 6 controls. Regarding the TNF gene, we analyzed the polymorphism rs1800629 that was present in 6 cases and 4 controls. As last we studied the polymorphism 1800872 of the IL-10 gene: 10 women of case group resulted positive for this mutation and 5 women from of control group. No statistically significant difference was found between the group of the cases and that of controls, but we found different percentages of two of the polymorphisms in the gene ABCB1: 75% of women in the control group showed SNP rs2032582 and only 50% in the case group; SNP rs1045642 was present in 39% of women in the controls group vs 60% in the case group.

## 4. Discussion

Early diagnosis of preeclampsia is essential to be able to set an adequate treatment and prevent the most serious complications of this disease such as eclampsia and intrauterine growth restriction. For this reason, the knowledge of the pathogenetic mechanisms underlying this condition and the identification of reliable hallmarks for early diagnosis is crucial.

We tried to identify an allele variant specifically associated with preeclampsia that could be an interesting marker to identify patients who present an increased risk of developing severe preeclampsia at an early stage.

Since we compared the pregnancy to a transplant and one of the targets of preeclampsia is the kidney, we decided to consider the genetic polymorphisms involved in the mechanisms of kidney transplant rejection. These SNPs are involved in immune response, in particular they are targets of the immunosuppressive therapy in kidney transplant [5,6,7].

In this study we analyzed five single nucleotide polymorphisms: one SNP of the IL-10 gene, one SNP of TNF gene and three SNPs of ABCB1 gene. These SNP were implicated in the response to immunosuppressive therapies in acute renal transplant rejection [5,7,8,9,10].

We analyzed their allelic and genotypic frequencies in pregnant women in order to determine a possible association with preeclampsia. Despite our study didn’t find any statistically significant results, the difference found in two of the analyzed polymorphisms of the ABCB1 gene (SNP rs2032582 and SNP rs1045642) could be interesting.

Furthermore, the ABCB1 gene encodes a P-glycoprotein belonging to the superfamily of the ATP-binding cassette (ABC) transporters which make up the bloodbrain barrier and also the placental barrier. This protein has protective functions as it participates in the elimination of metabolites, toxins and some drugs [11]. Number of polymorphisms of P-glycoprotein gene has been analyzed to determine possible effects on function of placental barrier [12,13]. This protein is a transmembrane efflux pump, which may expel various xenobiotics, and so immunosuppressive drugs like those used for the prevention of acute rejection after kidney transplant [7]. In previous studies on acute rejection after renal transplantation, an increased ABCB1 activity related to some polymorphisms of the gene caused a reduced intracellular exposure of T lymphocytes to the cyclosporine, an anti-rejection drug, which cannot explicate its inhibitory action and, because of this, it was connected with acute rejection event [14].

According to the study of Scalzotto et al. [10] the SNP rs1045642 showed greater involvement in the immune tolerance mechanisms of the transplant. In fact, it is more frequent in patients with acute renal transplant rejection and also, as shown in our study, in patients with preeclampsia. As regards the SNP rs2032582, our results are in contrast with the study just mentioned. Certainly, a larger sample will help us to better understand the real implication of these polymorphisms in the pathogenesis of preeclampsia.

As far as fetal outcomes are concerned, the data on prematurity should be highlighted: in our population sixteen out of twenty babies were born before 37 weeks in the preeclamptic group, while in the physiological pregnancy group all babies were born at term. This data is in agreement with the literature pointing out preeclampsia as a risk factor for prematurity [15,16].

Although our analysis did not report statistically significant differences between the two groups regarding the polymorphisms of the analyzed genes, our work may be helpful for future research to guide the choice of polymorphisms possibly implicated in the pathogenesis of preeclampsia.

We must also emphasize that it is the first study investigating these genes as possible risk factors implicated in the pathogenesis of preeclampsia.

As we know, preeclampsia has a multifactorial pathogenesis [17], and the role of genetics should not be forgotten but rather research should be implemented.

One of the limitations of our study is the small sample analyzed and this fact could condition the lack of significance. Probably expanding the sample of subjects analyzed we could obtain statistically significant results for the two polymorphisms just mentioned.

Furthermore, one of the advantages of our study is the use of a matched-with-age control group which was able to reduce the bias of age.

Polymorphisms’ analysis is an expanding field and future research could focus on this analysis not only to identify earlier susceptible subjects to the development of this disease but also to personalize therapies and to predict which patients will not respond to conventional therapies.

## Figures and Tables

**Table 1:** Diagnostic criteria of preeclampsia [1].

**Diagnostic Criteria**	**Brief Explanation**
new-onset hypertension	systolic blood pressure of 140 mmHg or more or diastolic blood pressure of 90 mmHg or more on two occasions at least 4 h apart after 20 weeks of gestation
And new-onset proteinuria	300 mg or more per 24 h urine collection or protein/creatinine ratio of 0.3 mg/dL or more
**Diagnostic Criteria in the Absence of Proteinuria**	**Brief Explanation**
thrombocytopenia	platelets count less than 100,000 × 10^9^/L
renal insufficiency	serum creatinine concentrations greater than 1.1 mg/dL or a doubling of the serum creatinine concentration in the absence of other renal disease
impaired liver function	elevated blood concentrations of liver transaminases to twice normal concentration
pulmonary edema	Evidence of pulmonary edema
new-onset headache	unresponsive to medication and not accounted for by alternative diagnoses
visual symptoms	flashing lights, auras, light sensitivity, blurry vision or spots

**Table 2:** Diagnostic criteria of severe preeclampsia [1].

Affected Organs and Systems	Diagnostic Criteria
Blood pressure	Systolic blood pressure of 160 mmHg or more or diastolic blood pressure of 110 mm Hg or more on two occasions at least 4 h apart
Platelets	Platelets count less than 100,000 × 10^9^/L
Liver	Abnormally elevated blood concentrations of liver enzymes and severe persistent right upper quadrant or epigastric pain unresponsive to medication and not accounted for by alternative diagnoses
Kidney	Serum creatinine concentration more than 1.1 mg/dL or a doubling of the serum creatinine concentration in the absence of other renal disease
Lung	Pulmonary edema
Nervous system	New on-set headache unresponsive to medication and not accounted for by alternative diagnoses
Sight	Visual disturbances

**Table 3:** Characteristics of the primers for each SNP.

Gene	SNP	Forward Primer (5′ → 3′)	Reverse Primer (5′ → 3′)	Length, bp
IL-10	rs1800872	GCGTGTTCCTAGGTCACAGT	ACTCTTACCCACTTCCCCCA	369
TNF	rs1800629	GCCAAGACTGAAACCAGCAT	TTGGGGACACACAAGCATCA	515
ABCB1	rs2032582	GTCCAAGAACTGGCTTTGCT	GCATGAGTTGTGAAGATAATA	446
ABCB1	rs1128503	CAACATCAGAAAGATGTGCAA	TGAGTTGGCCATCTATCCACC	615
ABCB1	rs1045642	AGTGTGGCCAGATGCTTGTA	CTGCCTACCACATGCATACAT	593

ABCB1, adenosine triphosphate-binding cassette sub-family B member 1; bp, base pair; IL-10, interleukin-10; SNP, single-nucleotide polymorphism; TNF, tumor necrosis factor.

**Table 4:** Main results.

Item Analyzed	CASES	CONTROLS	*p*-Value
Mean age	35.7 Std. dev. 4.96	36.2 Std. dev. 3.76	0.8029
Mean body mass index (BMI)	24.028 Std. dev 4.24	26.004 Std. dev. 7.60	0.4031
rs1800872 polymorphism in the gene IL10 (%)	50.00	58.82	0.706
rs1800629 polymorphism in the gene TNFα (%)	40.00	33.33	1.000
rs2032582 polymorphism in the gene ABCB1 (%)	50.00	75.00	0.231
rs1128503 polymorphism in the gene ABCB1 (%)	60.00	55.00	1.000
rs1045642 polymorphism in the gene ABCB1 (%)	60.00	38.89	0.433

## Data Availability

The data presented in this study are available on request from the corresponding author.

## Abbreviations

ABCB1 gene	adenosine triphosphate-binding cassette sub-family B member 1
SNP	single nucleotide polymorphism
MDR1 gene	multi drug reactivity 1 gene
IL10	interleukin 10
TNFα	tumor necrosis factor α
TH1	T helper phenotype 1
TH2	T helper phenotype 2
BMI	body mass index
Bp	base pair
